# Antagonistic Interactions in Mitochondria ROS Signaling Responses to Manganese

**DOI:** 10.3390/antiox12040804

**Published:** 2023-03-25

**Authors:** Jolyn Fernandes, Karan Uppal, Ken H. Liu, Xin Hu, Michael Orr, ViLinh Tran, Young-Mi Go, Dean P. Jones

**Affiliations:** 1 Section of Neonatal-Perinatal Medicine, Department of Pediatrics, College of Medicine, University of Oklahoma Health Sciences Center, Oklahoma City, OK 73104, USA; jolyn-fernandes@ouhsc.edu; 2 Division of Pulmonary, Allergy, Critical Care and Sleep Medicine, Department of Medicine, Emory University, Atlanta, GA 30322, USA

**Keywords:** mitochondria, antioxidants, oxidative phosphorylation, redox regulation, exposome, integrated omics, neurotoxicant, network of genes and metabolites

## Abstract

Antagonistic interaction refers to opposing beneficial and adverse signaling by a single agent. Understanding opposing signaling is important because pathologic outcomes can result from adverse causative agents or the failure of beneficial mechanisms. To test for opposing responses at a systems level, we used a transcriptome–metabolome-wide association study (TMWAS) with the rationale that metabolite changes provide a phenotypic readout of gene expression, and gene expression provides a phenotypic readout of signaling metabolites. We incorporated measures of mitochondrial oxidative stress (mtOx) and oxygen consumption rate (mtOCR) with TMWAS of cells with varied manganese (Mn) concentration and found that adverse neuroinflammatory signaling and fatty acid metabolism were connected to mtOx, while beneficial ion transport and neurotransmitter metabolism were connected to mtOCR. Each community contained opposing transcriptome–metabolome interactions, which were linked to biologic functions. The results show that antagonistic interaction is a generalized cell systems response to mitochondrial ROS signaling.

## 1. Introduction

Technological advances to process large biological data sets are projecting a dynamic and critical change into the global analysis of environmental stressors and disease [1]. These advances transform mechanistic research by enabling the study of effects on multiple targets as determinants of outcome rather than limiting hypotheses to singular determinants. In human, murine and cell studies, this multi-targeted approach reveals candidate regulatory determinants with intervention potential. Integrated omics studies show that a synergistic stress response hub was identified for combined fungicide (maneb) and herbicide (paraquat) exposure linked to Parkinson’s disease. In this published study, PPAR-γ and NRF2 genes were associated with protective responses, while pro-apoptotic genes were associated with damage response. These adverse and beneficial outcomes were interpreted based on known gene functionality [2] and not by measured functional parameters within the cell. Other network-based studies showed immune networks of genes and metabolites that correlate with later adaptive response to herpes zoster vaccination [3]; that network structures connect inflammation to lung metabolomics following viral infection [4,5]; and that redox proteomics in cadmium toxicity links fatty acid metabolites to respective mitochondrial fatty acid enzymes and transport systems [6,7]. In principle, such approaches can disentangle complex relationships among beneficial and harmful signaling responses to environmental exposures, an emerging need for exposome research [8,9,10].

Mitochondrial connections with other cell functions are especially important because mitochondria are susceptible to environmental agents and are essential for genetic and metabolic signaling to maintain and integrate energy, fatty acid and amino acid metabolism [11,12]. Although often conceptualized as occurring through discrete pathways and studied by isolating and targeting specific mitochondrial sites, stressors involving reactive oxygen species (ROS) impact multiple targets within and outside of mitochondria. This results in multiple signals, some with opposing beneficial and adverse effects. Such an antagonistic interaction is well-recognized in genetics [13,14] and aging [15,16,17,18], but has been little studied in exposome research.

Manganese (Mn) is an essential trace element with characteristics suitable to study antagonistic interactions from environmental exposures. Mn is required at low doses as a co-factor for the antioxidant mitochondrial protein superoxide dismutase-2 (SOD2), as well as by many other enzymes. Mn at high doses causes neurotoxicity (Manganism, neurodevelopmental defects, intellectual cognitive deficits, parkinsonian symptoms) due to occupational and environmental exposures [19,20,21,22,23]. Within the cell, Mn undergoes redox reactions and stimulates mitochondrial oxidant production [24,25,26,27]. Mn is similar in size and charge to other metals, such as iron and calcium, leading to alteration in their functions [28,29,30] and inducing further oxidative stress. Mn also interacts with electron transport chain complexes and impedes mitochondrial oxidative phosphorylation [24,27,31,32], increasing mitochondrial oxidative stress. Previous studies from our group have shown linear responses of the external trigger Mn directly on genes [33] and metabolites [34] separately, without considering both linear and nonlinear functional parameters as intermediate signaling and their role in gene–metabolite interactions.

To test for antagonistic interactions, we used a data-driven approach with xMWAS [12,35] to integrate metabolomics and transcriptomics with functional measures of mitochondrial activities in human neuroblastoma SH-SY5Y cells treated with increasing Mn concentrations for 5 h. Raw data from three previously published distinct studies [27,33,34] were used to identify mitochondrial-cellular signaling (MCS) subnetwork structures in response to Mn (Figure S1). The resulting ranked correlations of metabolites and transcripts with functional parameters such as cellular Mn, cellular thiols, mitochondrial ROS (mtROS), mitochondrial H_2_O_2_ (mtH_2_O_2_), SOD2 activity, mitochondrial basal oxygen consumption rate (OCR) and proton leak OCR (mtOCR) were used with community detection tools and targeted analyses to reveal subnetwork structures that mapped to known adaptive and adverse toxicologic responses. The results establish that the causative agent Mn affects functional responses, resulting in adverse and beneficial mitochondria-cell signaling mechanisms that occur simultaneously and provide a generalizable approach to study antagonistic interactions at a systems level.

## 2. Materials and Methods

### 2.1. Cell Culture, Mn Dose Treatment, Big Data Acquisition

Raw data (mitochondrial cellular parameters, metabolomics, RNA seq) from three previously published distinct studies [27,33,34] were used with xMWAS to identify MCS subnetwork structures in response to a Mn dose in human neuroblastoma cells (SH-SY5Y). The study design is provided in a schematic (Figure S1).

Cellular Mn concentrations were measured by graphite furnace atomic absorption spectroscopy (GFAA) (Thermo Scientific, Waltham, MA, USA, iCE3000 Series), total cellular thiols were measured using Ellman’s reagent, mitochondrial function assay was measured using WST-1(Roche), global mitochondrial oxidant production was measured using Mitotracker Red CM-H2XRos (Thermo scientific), mitochondrial H_2_O_2_ production was measured using mitochondrial peroxy yellow 1 (MitoPY1) (Tocris Biosciences, Minneapolis, MN, USA) and mitochondrial respiration was analyzed using a Seahorse Bioscience XF96 extracellular flux analyzer (North Billerica, MA, USA). Median value for each functional parameter at each Mn dose was obtained, and the resultant functional output matrix was used in downstream omics integration analysis (Figure S1).

Metabolomics analyses with three technical replicates were performed with ultra-high-resolution mass spectrometry with hydrophilic interaction liquid chromatography (HILIC) [Accucore HILIC 100 × 2.1 mm columns] in the positive ionization mode and with reverse-phase C18 liquid chromatography [Targa C18 (2.1 mm × 50 mm × 2.6 μm), Higgins Analytical] in negative ionization mode on a Q-Exactive HF (Thermo Fisher Scientific, Waltham, MA, USA). Data were extracted using apLCMS [36] and xMSanalyzer [37] to yield datasets with mass to charge (*m*/*z*), retention time (RT, s) and intensity for each mass spectral feature for all samples. The data were further filtered so 80% of all the samples had an intensity, quantile normalized and log2 transformed. Median value for each Mn dose was obtained, and the resultant metabolome data matrix was used in downstream omics integration analysis (Figure S1).

RNA extraction, RNA-Seq analysis and further data processing to obtain transcriptomics read counts has been previously described [33]. Raw data are made accessible to the public at Geo-NCBI with accession GSE129336. Briefly, total RNA was extracted using miRNeasy Mini Kit (Qiagen) and processed for RNA-Seq analysis following quality control checks for each sample. The platform used for sequencing was the Illumina HiSeq 2500. The data were normalized by variance stabilization transformation as implemented in the DESeq2 R/Bioconductor package [38]. Median value for each Mn dose was obtained, and the resultant transcriptome data matrix was used in downstream omics integration analysis (Figure S1).

### 2.2. Integrated Multi-Omics Network Analysis

The MCR dataset, HRM dataset and transcriptome dataset for Mn dose response were integrated using an xMWAS *v*0.54 tool based on the partial least squares (PLS) regression method for pairwise data integration [12]. xMWAS is a web-based data integration software used for differential network analysis of two or more omics platforms with biochemical responses available at https://github.com/kuppal2/xMWAS accessed on June 2020. The 3 datasets used as input for xMWAS included data for 5 Mn doses along with control and consisted of—1. *Mitochondrial-cellular parameters* with 7 measures (x) including cellular responses such as intracellular Mn concentration (Mn), total cellular thiols (thiols) and mitochondrial-specific responses such as ROS production (mtROS), H_2_O_2_ production (mtH_2_O_2_), SOD2 activity, basal oxygen consumption rate (bOCR) and proton leak dependent oxygen consumption rate (pOCR) measures; 2. *Metabolome data matrix* (y) with 6296 metabolic features; 3. *Transcriptome data matrix* (z) with 19,965 transcripts (Figure S1). Correlation threshold criteria for determining significant associations were set at (|r| > 0.8) and *p* < 0.05. Community detection was also performed using the multilevel community detection method [39], wherein a community consists of tightly connected biomolecules, herein genes, metabolites and the MCR parameters. The resultant multi-data integrative network provided by xMWAS allowed for identification and visualization of associations between mitochondrial functional outputs, genes and metabolites.

### 2.3. Pathway Analysis for Selected Metabolic Features and Genes

Metabolic features, selected based on the correlation threshold criteria, were subjected to permutation testing (*p* < 0.05) in pathway enrichment analysis using mummichog *v*1.0.9 [40]. Only metabolic pathways including greater than 3 overlap sizes were selected for further analysis. Genes selected based on the correlation threshold criteria were examined for enrichment of known biological processes in human species using DAVID *v*6.8, the Database for Annotation, Visualization and Integrated Discovery. The functional classification tool used generated a gene-to-gene similarity matrix based on shared functional annotation by using over 75,000 terms from 14 functional annotation sources [41].

### 2.4. Metabolite Annotation and Identification

Metabolites selected based on pathway analysis were annotated using an in-house library of confirmed metabolites and xMSannotator *v*1.3.2 [42] with Human Metabolome Database *v*3.5. Level 1 metabolite identification by the criteria of Schymanski et al. (Schymanski et al., 2014) was performed by co-elution relative to authentic standards and ion dissociation mass spectrometry. Additional annotations (levels 3, 4 and 5 metabolite identification criteria by [43]) were made using medium to high confidence scores from xMSannotator and using the KEGG (Kyoto Encyclopedia of Genes and Genomes) [44] and HMDB (Human Metabolome Database) [45] databases at *m*/*z* match of 10 ppm tolerance. Confidence scores for annotation by xMSannotator were derived from a multistage clustering algorithm based on correlation, co-elution, network modularity, and adducts/isotopes patterns. Additional information on metabolites discussed in the figures is plotted in Table S9.

### 2.5. Subcellular Fractionation and Western Blotting

To examine nuclear translocation of p65 NF-κB by Mn and Mn + MitoQ, cells were exposed to Mn doses (0, 10 and 100 μM for 5 h) with or without prior treatment with 1 μM MitoQ or vehicle control for 30 min. Post-exposure, cells were isolated for subcellular fractionation using a nuclear and cytoplasmic extraction kit (Thermo Scientific, Rockford, IL, USA). Isolated fractions were confirmed by Western blotting using antibodies against p65 NF-κB. Controls were determined by probing nuclei for lamin A/C. All antibodies were obtained from Cell Signaling Technology, Boston, MA, USA. An IRDye 800 conjugated affinity purified anti-rabbit (Rockland Immunochemicals, Gilbersville, PA, USA) or Alexa-Fluor-680-conjugated anti-mouse (Invitrogen, Waltham, MA, USA) was used as secondary antibody. Bands were visualized using an Odyssey scanner (Li-Cor Biosciences, Lincoln, NE, USA) and Odyssey 2.1 software (Li-Cor, Lincoln, NE, USA) and quantified using ImageJ v1.48.

All statistical analyses, boxplots and heatmaps were generated in R *v*3.2.3. Schematic figures were created using online software at BioRender.com. Abbreviations used throughout the text are provided in Supplementary Text S1.

## 3. Results

**Data-driven integration of mitochondrial activity with transcriptome and metabolome.**

**Figure 1:**
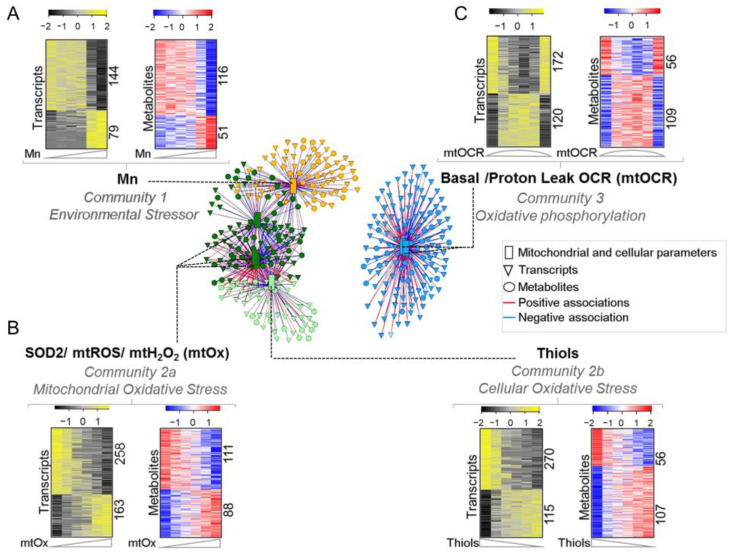
**Data-driven integrative analysis defined 3 communities with transcriptome and metabolome interaction with mitochondrial-cellular parameters [MCP] in response to Mn dose.** Three major communities generated through PLS regression method for pairwise data integration using xMWAS v0.54 at |r| > 0.9. (**A**) Community 1 (yellow)—environmental stressor subnetwork comprising intracellular Mn positively associated with 79 genes and 51 metabolites and negatively associated with 144 genes and 116 metabolites. (**B**) Community 2 (green)—oxidative stress subnetwork further bifurcated into two sub-communities at |r| > 0.8—communities 2a and 2b. Community 2a (dark green)—mitochondrial oxidative stress (mtOx) subnetwork community comprising SOD2, mtROS and mtH_2_O_2_ positively associated with 163 genes and 88 metabolites and negatively associated with 268 genes and 111 metabolites. Community 2b (light green)—cellular oxidative stress subnetwork comprising total cellular thiols positively associated with 270 genes and 56 metabolites and negatively associated with 115 genes and 107 metabolites. (**C**) Community 3 (blue)—oxidative phosphorylation subnetwork (mtOCR) comprising basal OCR and proton leak dependent OCR, positively associated with 120 genes and 109 metabolites and negatively associated with 172 genes and 56 metabolites. Seven MCPs are represented as rectangles, genes as triangles and metabolites as circles. Red edges represent positive, while blue represents negative associations with their respective MCPs. |r| > 0.8, *p* < 0.05.

Partial least squares (PLS) regression in xMWAS provided ranked correlations for the interactions of seven mitochondrial and cellular parameters (MCP) (Figure S1). From these correlations, community detection allowed visualization of three major communities linked to cellular Mn (Community one, Figure 1A), mitochondrial oxidative stress [mtOx (mtROS, mtH_2_O_2_, SOD2 activity)] and total cellular thiols (Community two; Figure 1B), and mitochondrial oxygen consumption rate [mtOCR (basal OCR and proton leak OCR)] (Community three; Figure 1C), which were preserved at increased stringency (|r| > 0.9; Figure 1, center). The composition of the communities at |r| > 0.8 included 480 metabolites and 947 genes (Figure S1), with genes and metabolites differing for Community one (Tables S1 and S2), Community two (Tables S3–S6) and Community three (Tables S7 and S8). Community two had two sub-clusters centered on mtOx (Community 2a; mtROS, mtH_2_O_2_ and SOD2 activity) and cellular thiols (Community 2b), which were largely negatively associated with each other.

From a top-down view, Community one included transcripts and metabolites most closely associated with cellular Mn, perhaps reflecting non-mitochondrial mechanisms of toxicity and homeostasis. The structures of Community two contained elements linked to mitochondrial oxidative stress, the main mechanism of cell toxicity. To validate this interpretation, we performed a targeted experiment with mitoQ, an inhibitor of mitochondrial oxidative stress, and found that NF-κB translocation to the nucleus, a well-established response to oxidative signaling, was blocked (see below) (Figure S2). Community two had opposing substructures for mitochondrial ROS (Community 2a) and cellular thiol content (Community 2b), consistent with known causal mechanisms in which thiols are oxidized in response to oxidative stress. Community three included elements more strongly associated with mitochondrial respiration than with Mn or oxidative stress. For every gene and metabolite, the direction of positive and negative correlation with the functional measure is provided as [+] and [−], respectively, throughout the text. Directionality is also provided for the rest of genes and metabolites represented by correlation coefficient, presented in the supplementary tables as +|r| or −|r|.

**Community one: Cell Mn was associated with biological processes for biosynthesis, morphogenesis, amino acid metabolism, differentiation and apoptosis.**

**Figure 2:**
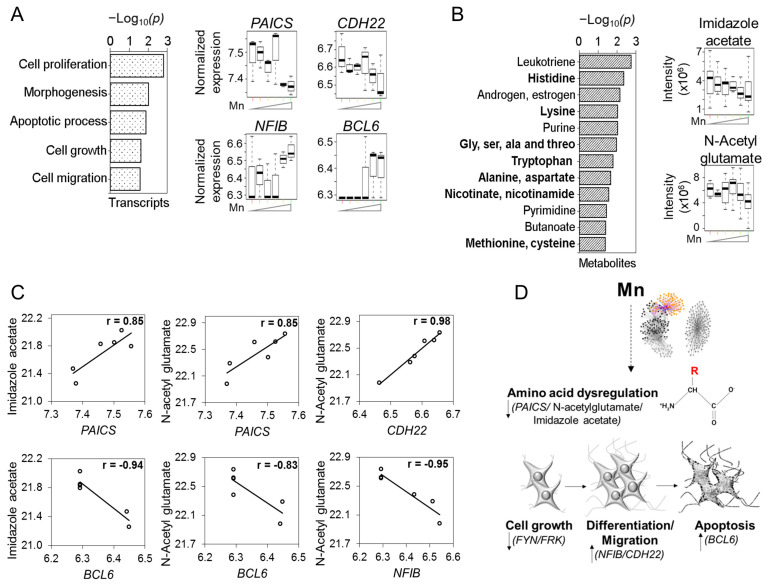
**Community 1: environmental stressor subnetwork associated with signaling markers for biosynthesis, morphogenesis, amino acid metabolism, differentiation and apoptosis**. Pathway enrichment analysis of genes and metabolites associated with cellular Mn subnetwork were studied. (**A**) Top pathways enriched by Mn-dependent genes at *p* < 0.05, represented as −log_10_(*p*) Expression of Mn-dependent individual genes from these enriched pathways presented as boxplots include genes involved in amino acid metabolism—PAICS; morphogenesis—CDH22; differentiation—NFIB and apoptosis—BCL6. (**B**) Mn-dependent metabolites enriched in amino acid and energy related pathways (bold) at *p* < 0.05 depicted as −log_10_(*p*). Raw intensity of individual metabolites from these enriched pathways presented as boxplots include amino acid metabolites—imidazole acetate and N-acetyl glutamate. (**C**) Antagonistic interactions of genes and metabolites in cellular Mn-dependent cluster. (**D**) Schematic illustrating cellular Mn-associated key drivers of cell growth, differentiation, migration and apoptosis through amino acid dysregulation. Detailed information on genes and metabolites from Community 1 provided in Tables S1, S2 and S9. |r| > 0.8, *p* < 0.05.

We examined each community for gene–metabolite associations that could be traced to signaling mechanisms. Previous research showed adaptive responses to non-toxic Mn exposure in amino acid metabolism and protein secretion pathways, while toxic Mn exposures disrupted fatty acid and energy metabolism [33,34]. Integrated TMWAS showed that transcripts for purine biosynthesis (*PAICS*, phosphoribosylaminoimidazole carboxylase) [−] and morphogenesis and development (*CDH22*, cadherin 22, type 2) [−] [46,47] (Figure 2A) varied positively with imidazole acetate and *N*-acetylglutamate [−] (Figure 2B), while transcripts regulating differentiation (*NFIB*, nuclear factor I/B) [+] and apoptosis (*BCL6*) [+] [48,49] varied negatively with imidazole acetate [−] and *N*-acetylglutamate [−] (Figure 2C). The gene–metabolite pair is based on highest correlation values between the two and their enrichment in the pathway analysis (Figure 2C). The results show an apparent antagonistic interaction associated with imidazole acetate and *N*-acetylglutamate and illustrate that Community one consists of opposing transcriptome–metabolite interactions (Figure 2D).

At the transcriptome level, Community one showed that cellular Mn was associated with changes in 223 genes (Figure 1A), including 176 mRNA, 31 long non-coding (lncRNA), 15 miRNA (microRNA) and 1 sncRNA (Y RNA) (Table S1). Biologic processes associated with mRNAs included cell proliferation, anatomical morphogenesis, growth regulation, cell migration and apoptosis (Figure 2A). Potentially adverse changes included the decrease in *PAICS* and *CDH22* as well as *FYN* and *FRK* members of Src family of tyrosine kinases functioning in cell growth and proliferation [50,51] (Table S1).

Intracellular Mn was associated with changes in 167 metabolites (Figure 1A and Table S2), which showed broad impact by pathway enrichment analysis on amino acid pathways (His; Lys; Gly, Ser, Ala and Thr; Trp; Ala and Asp; Met and Cys) and the nicotinamide pathway (Figure 2B). Imidazole acetate is involved in His metabolism, and *N*-acetylglutamate is an activator of carbamoyl phosphate synthase I in the urea cycle (Figure 2B) [52,53]; both were decreased by Mn. Hexose-bisphosphate, a precursor for glycolysis and the pentose phosphate pathway, and riboflavin, involved in energy pathways, were also decreased by Mn (Table S9).

**Community two: Mitochondrial oxidative stress (mtOx) was associated with inflammation and fatty acid metabolism.**

**Figure 3:**
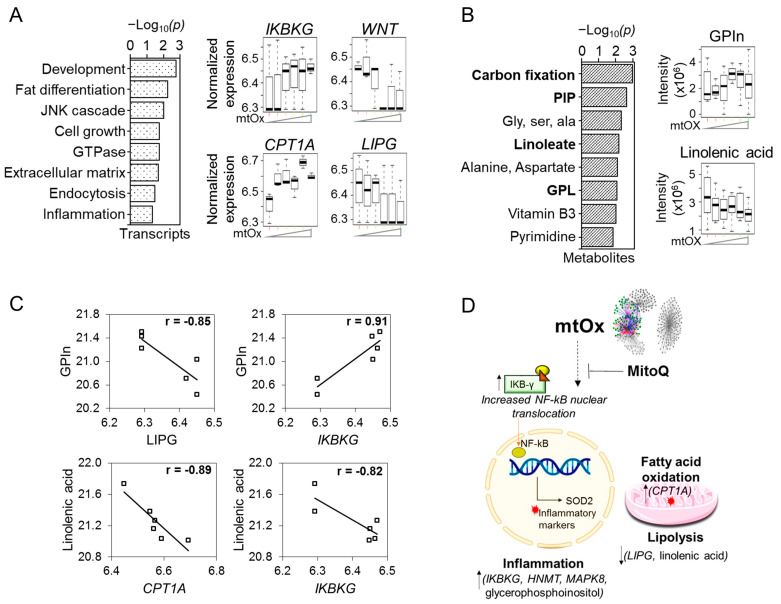
**Community 2a: mitochondrial oxidative stress subnetwork associated with signaling markers for inflammation, lipid and fatty acid metabolism**. Pathway enrichment analysis of genes and metabolites associated with mitochondrial oxidative stress (mtOx) subnetwork was studied. (**A**) Top pathways enriched with mtOx-dependent genes at *p* < 0.05, represented as −log_10_*P*. Expression of individual genes associated with mtOx from these enriched pathways presented as boxplots include genes involved in inflammation and NF-κB activation—IKBKG; in neuroprotection—WNT1; fatty acid oxidation—CPT1A and lipid metabolism—LIPG. (**B**) Metabolite-enriched lipid metabolism pathways such as carbon fixation, phosphotidyl inositol phosphate, linoleate, glycerophospholipid and fatty acid metabolism (bold) at *p* < 0.05 depicted by −log_10_*P*. Raw intensity of individual metabolites from these enriched pathways presented as boxplots include pro-inflammatory metabolite—glycerophosphoinositol; and fatty acid metabolite—linolenic acid. (**C**) Antagonistic interactions of genes and metabolites in mitochondrial oxidative stress cluster. (**D**) Schematic illustrating SOD2-, mtH_2_O_2_- and mtROS-associated key drivers of inflammation and dysregulation of lipid metabolism and fatty acid oxidation. The mtOx intensity on the *x*-axis is indicative of Mn exposure low–med–high from left to right. Detailed information on genes and metabolites from Community 2a provided in Tables S3, S4 and S9. |r| > 0.8, *p* < 0.05.

Mitochondrial oxidative stress represents a key mechanism in neurotoxicity caused by excess Mn. In the xMWAS, all three mitochondrial oxidative stress measures (mtROS, mtH_2_O_2_ and SOD2) were tightly correlated in a sub-community, Community 2a. Cell thiol loss, a downstream consequence of increased ROS, partially separated as Community 2b with negative associations from Community 2a. Examination of the TMWAS for Community 2a showed that the proinflammatory transcript *IKBKG* (inhibitor of kappa light polypeptide gene enhancer in B-cells, kinase gamma) [+] encoding a protein that activates NF-κB [54] (Figure 3A) was positively associated with the anti-inflammatory glycerophosphoinositol [+] (Figure 3B,C). As a proof of concept experiment to show that data-driven associations without using metabolic perturbations can be validated in a system, additional experiments were conducted. IKBKG (NEMO) encodes a protein that activates NF-kB, which leads to NF-kB translocation to the nucleus and induces inflammatory signal [55]. Our current network analysis demonstrates Mn-dependent mt-Ox measures are positively associated with IKBKG gene, suggesting that Mn-dependent mitochondrial ROS-production could lead to NF-kB translocation. To demonstrate this, Mn exposed cells were treated with and without MitoQ, a mitochondrial targeted antioxidant, to decrease mitochondrial oxidative stress. Cells exposed to Mn indeed showed NF-kB translocation, which was further blocked by Mito-Q. These results demonstrate and validate our network analysis interpretation that Mn-dependent mitochondrial ROS-production indeed leads to NF-kB translocation and potential inflammatory signaling (Figure S2). In addition to IKBKG, transcript data for other NF-kB components are presented (Figure S3). Thus, network analysis can reveal hidden relationships just by functional measures integrated with omics data.

In the opposite direction, the essential fatty acid linolenate [−] associated negatively with *IKBKG* [+] (Figure 3C, Table S4). Opposite effects were also seen when comparing glycerophosphoinositol [+] with the phospholipase gene *LIPG* [−] (Figure 3C, Table S4). *CPT1A*, encoding a key protein to support mitochondrial fatty acid oxidation, was increased with mtOx. Thus, pro-inflammatory processes were accompanied by opposite effects on fatty acid and lipid metabolism.

In addition to the general pattern of increased inflammatory gene transcripts with mtOx, including *IKBKG* (Figure 3A), *MAPK8* (involved in JNK signaling pathways) [+] and *HNMT* (functioning in histamine metabolism) [+] (Table S3), other transcripts in Community 2a (Table S3) were negatively associated with mitochondrial measures of oxidative stress (Figure 1C). These included decreased *WNT1*, a transcript for a protein that inhibits inflammation and is neuroprotective [56] (Figure 3A).

**Figure 4:**
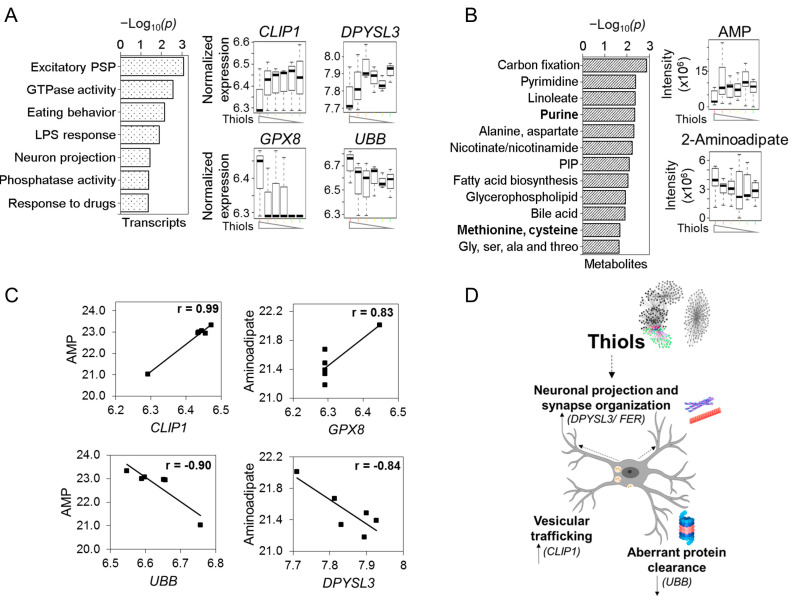
**Community 2b: cellular oxidative stress subnetwork associated with signaling markers for neuronal projection, vesicular trafficking and aberrant protein clearance**. Pathway enrichment analysis of genes and metabolites associated with total cellular thiols subnetwork was studied. (**A**) Top pathways enriched with total cellular thiol dependent genes at *p* < 0.05, represented as −log_10_*P.* Expression of individual genes associated from these enriched pathways presented as boxplots include genes regulating synaptic and vesicular trafficking and axonal guidance, such as CLIP1 and DPYSL3; genes involved in ER ROS clearance—GPX8 and aberrant protein clearance—UBB. (**B**) Cellular thiol-associated metabolites enriched in pathways similar to Community 2b (mtOx cluster) except for methionine and cysteine and purine pathways (bold) at *p* < 0.05 depicted by −log_10_*P*. Raw intensity of individual metabolites from these enriched pathways presented as boxplots include purine metabolism—AMP and neurogenesis-related metabolite—α-aminoadipate. (**C**) Antagonistic interactions of genes and metabolites in cellular thiols cluster. (**D**) Schematic illustrating total cellular thiol-associated key drivers of neuronal projection, synapse organization, vesicular trafficking and aberrant protein clearance. The thiol intensity on the *x*-axis is indicative of Mn exposure low–med–high from left to right. Detailed information on genes and metabolites from Community 2b provided in Tables S5, S6 and S9. |r| > 0.8, *p* < 0.05.

Community 2b was centered on cell thiols, and elements within this subcommunity were frequently associated negatively with elements in Community 2a. TMWAS for Community 2b also included opposing responses, such as for *CLIP1* (CAP-Gly domain containing linker protein 1) [+], encoding a protein involved in microtubule dynamics and vesicular trafficking, and *UBB* (ubiquitin B) [−], functioning in ATP-dependent aberrant protein clearance (Figure 4A), varied in opposite directions in association with the nucleoside, AMP (Figure 4B, Table S6). *GPX8* (glutathione peroxidase 8) [−], encoding a protein for the elimination of H_2_O_2_ from the ER, and *DPYSL3* (dihydropyrimidinase-like 3) [+], encoding a protein functioning in cytoskeletal organization and axonal guidance, varied in opposite directions in association with α-aminoadipate (Figure 4C). *FER* (tyrosine kinase) [+], a gene supporting synapse organization and generation of excitatory postsynaptic currents, was also negatively associated with thiols (Table S5).

At the metabolomic level, pathway enrichment showed that the metabolites associated with cell thiols (Figure 1B, Table S6) were present in amino acid (e.g., alanine, glycine), niacin (nicotinate, nicotinamide) and purine and pyrimidine pathways (Figure 4B, Table S6). The overall results for Community 2 (Figure 4D), which represents the central toxicologic mechanism of Mn, therefore show patterns of antagonistic interaction within signals associated with increased ROS and within signals associated with loss of thiols.

**Community three: Mitochondrial respiration was associated with ion transport and neurotransmitter metabolism.**

**Figure 5:**
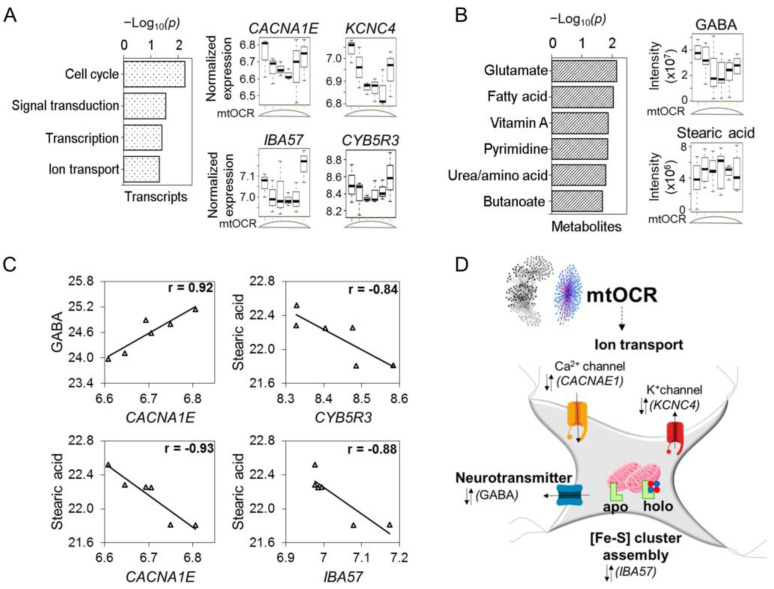
Community 3: oxidative phosphorylation subnetwork associated with signaling markers for ion transport, neurotransmitter metabolism and iron sulfur cluster assembly. Pathway enrichment analysis of genes and metabolites associated with basal OCR and proton leak dependent OCR (mtOCR) subnetwork were studied. (**A**) Top pathways enriched by mtOCR-dependent genes at *p* < 0.05, represented as −log_10_*P*. Expression of individual genes associated with biphasic mtOCR from these enriched pathways presented as boxplots include genes involved in ion transport channels for calcium, CACNA1E; potassium, KCNC4; iron sulfur cluster assembly gene, IBA57 and NADH-dependent reductase of redox cyclers, CYB5R3. (**B**) Top pathways representing mtOCR-dependent metabolites at *p* < 0.05, represented as −log_10_*P*. Raw intensity of individual metabolites from these enriched pathways presented as boxplots include neurotransmitter, GABA and long chain fatty acid, stearic acid. (**C**) Antagonistic interactions of genes and metabolites in mitochondrial oxidative phosphorylation cluster. (**D**) Schematic illustrating biphasic mtOCR-associated key drivers of ion transport, neurotransmitter metabolism and iron sulfur cluster assembly. The mtOCR intensity on the *x*-axis is indicative of Mn exposure low–med–high from left to right. Detailed information on genes and metabolites from Community 3 provided in Tables S7–S9. |r| > 0.8, *p* < 0.05.

Maintenance of mitochondrial function and cellular energetics is critical to protect against Mn toxicity. Basal OCR and proton leak OCR each had a biphasic response to Mn with stimulation at adaptive concentrations and decrease at toxic concentrations [27]. In xMWAS, genes and metabolites associated with basal and proton leak OCR clustered together in a tight mtOCR Community three (Figure 1C). TMWAS for Community three showed that when mtOCR was highest, the abundance of *CACNA1E* [−], a subunit of a high voltage activated calcium channel that regulates neurotransmitter signaling (Figure 5A), and the neurotransmitter, GABA [−] (Figure 5B, Table S8), were lowest. *CACNA1E* modulates neuronal firing patterns and is associated with encephalopathies [57,58,59]. In addition to neurotransmitter signaling, an imbalance in bioenergetics also leads to consumption of alternative fuel sources such as GABA to produce TCA cycle intermediates through the GABA shunt [60]. The decrease in *CACNA1E* abundance while the neurotransmitter GABA was also decreased with increased mitochondrial oxidative phosphorylation, suggests an antagonistic response in the regulation of neuronal firing, and neurotransmitter-related metabolic and intramitochondrial function. 

Negative associations were observed for the fatty acid, stearate and transcripts for iron sulfur assembly (*IBA57*) [−] and NADH reductase (*CYB5R3*, cytochrome b5 reductase 3) [−] (Figure 5C), showing that opposing responses integrate neurotransmitter systems and other homeostatic mechanisms (Figure 5D). Transcripts with protective functions that were positively associated with mtOCR included *UPP1* (uridine phosphorylase 1) [+], an ATP-dependent transcriptional regulator involved in pyrimidine salvage (Table S7). Transcripts that were negatively associated with the mtOCR included *KCNC4* [−], a potassium channel, and *SIRT6* [−], an NAD-dependent protein deacetylase involved in DNA repair (Table S7). Pathway enrichment analysis showed associations with multiple adaptive response pathways, such as cell cycle, intracellular signal transduction, transcription and ion transport (Figure 5A). Pathway enrichment analysis for metabolomics demonstrated glutamate as the topmost pathway, followed by fatty acid metabolism, vitamin A, pyrimidine, urea cycle and butanoate metabolism (Figure 5B and Table S9).

Overall, Community three associations linked to mitochondrial bioenergetic activity represent positive and protective response to increased Mn (Community one), as well as to oxidative stress (Community 2), but the results show that this occurs functionally through response structures with antagonistic signaling. Thus, the results from this community also support the conclusion that antagonistic interactions occur at sub-network levels downstream to the independent variable, Mn.

**Antagonistic patterns of non-protein coding transcripts associated with neurodegenerative diseases.**

**Figure 6:**
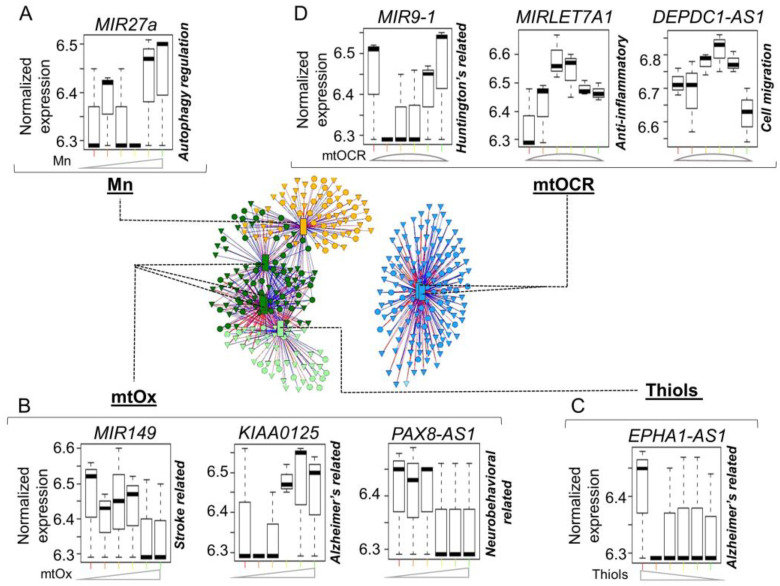
**Non-coding RNAs characterized in neurological disorders associated with mitochondrial-cellular parameters in response to Mn dose**. (**A**) Environmental stressor (Mn) subnetwork includes positively associated miRNA involved in regulation of autophagy-related protein PINK1, *MIR27a*. (**B**) Oxidative phosphorylation (mtOCR) subnetwork includes Huntington’s disease-related miRNA, *MIR9*-*1*; anti-inflammatory-related miRNA, *MIRLET7A1*; and cell migration-related lncRNA, *DEPDC1*-*AS1*. (**C**) Total cellular thiols subnetwork includes positively associated Alzheimer’s-related lncRNA, *EPHA1*-*AS1*. (**D**) Mitochondrial oxidative stress (mtOx) subnetwork includes stroke-related miRNA, *MIR149*, Alzheimer’s-related lncRNA, *KIAA0125* and neurobehavioral-associated lncRNA, *PAX8*-*AS1*. The intensity for functional measures on the *x*-axis is indicative of Mn exposure low–med–high from left to right. Details on all associated non-coding miRNAs and lncRNAs are provided Tables S1, S3, S5 and S6. |r| > 0.8, *p* < 0.05.

The capability to delineate opposing signaling processes raised the possibility that the integrative omics analysis using xMWAS described here could be used to evaluate antagonistic interaction in therapeutics. To test the feasibility of this concept, we considered Mn dosing as a model for a therapeutic treatment. We examined non-coding RNAs (ncRNA) associated with each community (highlighted in Tables S1, S3, S5 and S7) for ones involved in neurological dysfunction. ncRNAs, such as microRNA (miRNA) and lncRNA, are cellular regulators in various disease states including neurological dysfunction, cancer and oxidative stress [61,62]. Community-one-associated miRNA involved in regulation of autophagy-related protein PINK1 (PTEN-induced kinase 1, linked to Parkinson’s disease), *MIR27A*, increased with intracellular Mn (Figure 6A).

Community-2a-associated ncRNAs such as stroke-related *MIR149* and Alzheimer’s-related lncRNA, *KIAA0125*, increased with the increase in mtOx, and neurobehavioral-associated lncRNA, *PAX8*-*AS1*, declined with the increase in mtOx (Figure 6B). Community 2b was associated with Alzheimer’s-related lncRNA, *EPHA1*-*AS1*, which declined with the decrease in total cellular thiols (Figure 6C). Community-three-associated ncRNAs included Huntington’s disease-related *MIR9*-*1*, which was negatively associated with mtOCR, and anti-inflammatory-associated *MIRLET7A1* and cell migration lncRNA *DEPDC1*-*AS1*, which were positively associated with mtOCR (Figure 6D). The results show that application of xMWAS to interrogate antagonistic interactions has the potential to allow deconvolution of very complex biologic processes linked to ncRNAs and provide a strategy to identify disease-specific therapeutic targets, as well as monitor therapeutic response to intervention.

## 4. Discussion

Complex molecular interactions drive biological processes in response to environmental exposures. Utilizing an advanced multi-omics approach with data-driven bioinformatics analysis, the present study demonstrates that metabolomics and transcriptomics changes can be combined with cellular- and mitochondrial-specific responses to provide a systems view of underlying mechanisms relevant to neurotoxicity. We show that at multiple levels of network structure, the genes and metabolites involved with specific functional traits exhibit characteristics which we interpret as antagonistic interaction, a characteristic previously found for mitophagy [63], viral infections [64], antioxidant and redox systems and aging [18,65]. The present bioinformatics approach shows that opposing gene and metabolite responses occur directly related to the causal agent, Mn. Some of these antagonistic interactions reflect mechanisms of mitochondrial oxidative stress, the consequent oxidation of cell thiols and the downstream protective responses of mitochondrial bioenergetics.

Antagonistic interactions occur because Mn is a trigger that generates both positive and negative responses. Within the Mn network structure, genes such as *NFIB*, which is critical for cell survival, differentiation and migration in cortical and forebrain development [66], also include genes such as *BCL6* functioning in apoptosis. Complex interactions of antagonistic responses in a high voltage activated calcium channel controlling neuronal firing patterns that are associated with migraines and epileptic encephalopathies [57,58,59] associated with neurotransmitter, GABA, within the mitochondrial oxidative phosphorylation structure (mtOCR). These associations do not identify which is cause or effect; however, previous studies show that increasing calcium concentration directly inhibits mitochondrial oxidative phosphorylation in a dose- and time-dependent manner [67]. Other examples illustrate opposing associations that are not easily predicted. These include changes in an iron sulfur cluster assembly protein, IBA57, required for respiratory chain complexes I and II assembly [68], and cytochrome b5 reductase 3, required for cellular lipid homeostasis, [69,70], both of which negatively associated with lipid metabolites.

Multiple transcript–metabolite associations with oxidative stress (mtOx) have previously been implicated in mitochondrial dysfunction and neuroinflammation as mechanisms of neurological diseases, including Alzheimer’s disease [71,72] and progressive multiple sclerosis [73]. Some of the reactions that appear to represent antagonistic interaction may actually represent generalized disruption of cell function. For instance, decreased *LIPG* and *CYP27A1* could reflect a generalized dysregulation of lipid metabolism in response to mtOx. The causal relationship between phenotypes, however, will have to be determined. *CYP27A1* encodes a mitochondrial oxidase functioning in cholesterol metabolism. In addition, a decrease in linolenic acid and elevation in *CPT1A* could indicate a generalized disruption of lipid metabolism. Such an interpretation is consistent with *CYP27A1*-knockout mice and patients with CYP27A1 loss of function mutations, having hypertriglyceridemia in the brain characterized by behavior and motor dysfunction [74,75,76].

Similarly, opposing signals associated with the oxidation of cellular thiols could reflect generalized disruption of the redox proteome. Thiols are the main determinants of total antioxidant capacity of a cell [77], and previous studies show metals decrease cellular thiols and cause actin cytoskeletal remodeling [78]. The current results show an increase in several cytoskeletal genes, but do not show whether these are specific or non-specific responses to thiol oxidation. These include genes for collapsin response mediator protein 4 (CRMP4), a highly expressed protein in the adult nervous system regulating axonal guidance [79], FER, a cytoplasmic protein tyrosine kinase regulating microtubules [80], and CLIP1, a microtubule protein in vesicular trafficking and associated with intellectual disability [81]. Our findings build upon existent knowledge where similar pleiotropic signaling by reactive oxygen species has been previously described [82,83]

While systems biology research has emphasized the need for network approaches to understand complex systems, extensions to define opposing responses within subnetworks are limited. The communities identified in the present study were obtained from systematic variation in a single independent variable, Mn, and future studies will need to address how multiple interacting exposures impact response communities. In the present analysis, only Community one was directly related to Mn, and the other communities were associated with the dependent variables mtOx and mtOCR. Simultaneous exposures to other oxidants, antioxidants or respiratory fuels or inhibitors could alter these response structures. This exposure also includes the environment in which cells grow. Neuronal cells in the brain sense oxygen, leading to changes in its activity [84]. Response structures to Mn and dependent variables may be different under different oxygen-dependent conditions, such as hypoxia. Although not discussed, other associations between communities were present (see Supplemental Tables S1–S9). Additionally, associations below the feature selection threshold |r| > 0.8, were not visible in the data shown, which may constitute proteins encoded by mitochondrial DNA. The central conclusion that antagonistic interaction is a common feature of the complex systems response to excess Mn is supported by a wealth of evidence (Tables S1–S8). Targeted location of these system networks also becomes important as proteins are transferred from cytosol to mitochondria, especially mitochondrial proteins encoded by the nucleus or repair enzymes produced outside the mitochondria. Additional understanding will be derived from future identification of functions of miRNAs and lncRNAs, from experimental evidence in other cell types, integration with other omics platforms such as epigenetics and by establishing the interactions of pathways with Mn in primary versus immortalized cell lines, which will be needed for a global understanding of cellular and mitochondrial responses [85].

A strength of the approach is that the integration of multiple omics platforms can be used to improve mechanistic knowledge for any natural or anthropogenic environmental chemical exposure, drug or therapeutic intervention. The approach can be applied to any cell or animal model, as well as human studies. Recognition that opposing reactions are inherent within response structures aids in defining positive and negative responses, such as those that occur in bi-directional signaling between mitochondria and cell nuclei [86]. Additional research will be needed to understand which of these opposing reactions, whether functionally dependent or independent, are truly beneficial versus harmful responses, as distinguished from those that might represent rectifying pleiotropy, i.e., opposing responses that have evolved to allow homeostatic recovery after initial response to challenge. Such responses are well-known following inflammation, where resolvins are produced as silencers of proinflammatory signaling [87].

In conclusion, a data-driven bioinformatics analysis of mitochondria-cell phenotype in neuroblastoma cells dosed with Mn showed three major network communities, each of which had extensive evidence for opposing response structures, which we interpret as antagonistic interaction. While these can be segregated into individual responses, they likely work in concert to cause Mn-related neurological disorders. The approach using xMWAS to identify these opposing reactions is general and can be broadly applied to model systems and human health research. The implications for biomedical research are that all manifestations of health exposures are likely to include opposing detrimental and beneficial responses, which will need to be addressed at a systems level for effective intervention.

## Data Availability

Raw data from three previously published distinct studies [27,33,34] were used.

## Supplementary Materials

The following supporting information can be downloaded at: https://www.mdpi.com/article/10.3390/antiox12040804/s1, Figure S1: Integration of mitochondrial-cellular parameters (MCP) with metabolomics and transcriptomics data matrices. Figure S2: NF-κB nuclear signaling by Mn dose; Figure S3: Transcript data for other NF-kB components. Table S1: Genes associated with Community one (intracellular Mn) at |r| > 0.8 (*p* < 0.05). Table S2: Annotation of metabolic features associated with Community one (intracellular Mn) at |r| > 0.8 (*p* < 0.05). Table S3: Genes associated with Community 2a (mtOx) at |r| > 0.8 (*p* < 0.05). Table S4: Annotation of metabolic features associated with Community 2a (mtOx) at |r| > 0.8 (*p* < 0.05). Table S5: Genes associated with Community 2b (cellular thiols) at |r| > 0.8 (*p* < 0.05). Table S6: Annotation of metabolic features associated with Community 2b (cellular thiols) at |r| > 0.8 (*p* < 0.05). Table S7: Genes associated with Community three (mtOCR-mitochondrial oxygen consumption rate) at |r| > 0.8 (*p* < 0.05). Table S8: Annotation of metabolic features associated with Community three (mtOCR-mitochondrial oxygen consumption rate) at |r| > 0.8 (*p* < 0.05). Table S9: Detailed information on significant metabolites from pathways associated with MCPs from Figure 2, Figure 3, Figure 4 and Figure 5. Text S1: Abbreviations.

## References

[1] Manzoni C., Kia D.A., Vandrovcova J., Hardy J., Wood N.W., Lewis P.A., Ferrari R. (2018). Genome, transcriptome and proteome: The rise of omics data and their integration in biomedical sciences. Brief Bioinform..

[2] Roede J.R., Uppal K., Park Y., Tran V., Jones D.P. (2014). Transcriptome-metabolome wide association study (TMWAS) of maneb and paraquat neurotoxicity reveals network level interactions in toxicologic mechanism. Toxicol. Rep..

[3] Li S., Sullivan N.L., Rouphael N., Yu T., Banton S., Maddur M.S., McCausland M., Chiu C., Canniff J., Dubey S. (2017). Metabolic Phenotypes of Response to Vaccination in Humans. Cell.

[4] Chandler J.D., Hu X., Ko E.J., Park S., Lee Y.T., Orr M., Fernandes J., Uppal K., Kang S.M., Jones D.P. (2016). Metabolic pathways of lung inflammation revealed by high-resolution metabolomics (HRM) of H1N1 influenza virus infection in mice. Am. J. Physiol. Regul. Integr. Comp. Physiol..

[5] Bakker O.B., Aguirre-Gamboa R., Sanna S., Oosting M., Smeekens S.P., Jaeger M., Zorro M., Vosa U., Withoff S., Netea-Maier R.T. (2018). Integration of multi-omics data and deep phenotyping enables prediction of cytokine responses. Nat. Immunol..

[6] Go Y.M., Roede J.R., Orr M., Liang Y., Jones D.P. (2014). Integrated redox proteomics and metabolomics of mitochondria to identify mechanisms of cd toxicity. Toxicol. Sci..

[7] Go Y., Uppal K., Jones D.P. (2018). Central Mitochondrial Signaling Mechanisms in Response to Environmental Agents. Mitochondrial Dysfunction Caused by Drugs and Environmental Toxicants.

[8] Dennis K.K., Jones D.P. (2016). The Exposome: A New Frontier for Education. Am. Biol. Teach..

[9] Cortese-Krott M.M., Koning A., Kuhnle G.G.C., Nagy P., Bianco C.L., Pasch A., Wink D.A., Fukuto J.M., Jackson A.A., van Goor H. (2017). The Reactive Species Interactome: Evolutionary Emergence, Biological Significance, and Opportunities for Redox Metabolomics and Personalized Medicine. Antioxid. Redox Signal..

[10] Li-Pook-Than J., Snyder M. (2013). iPOP goes the world: Integrated personalized Omics profiling and the road toward improved health care. Chem. Biol..

[11] Chandel N.S. (2014). Mitochondria as signaling organelles. BMC Biol..

[12] Go Y.M., Fernandes J., Hu X., Uppal K., Jones D.P. (2018). Mitochondrial network responses in oxidative physiology and disease. Free Radic Biol. Med..

[13] Rodier F., Campisi J., Bhaumik D. (2007). Two faces of p53: Aging and tumor suppression. Nucleic Acids Res..

[14] Carter A.J., Nguyen A.Q. (2011). Antagonistic pleiotropy as a widespread mechanism for the maintenance of polymorphic disease alleles. BMC Med. Genet..

[15] Austad S.N., Hoffman J.M. (2018). Is antagonistic pleiotropy ubiquitous in aging biology?. Evol. Med. Public Health.

[16] Hashimoto M., Ho G., Takamatsu Y., Shimizu Y., Sugama S., Takenouchi T., Waragai M., Masliah E. (2018). Evolvability and Neurodegenerative Disease: Antagonistic Pleiotropy Phenomena Derived from Amyloid Aggregates. J. Parkinsons Dis..

[17] Park M., Wang M.C. (2018). Aging: Antagonistic Pleiotropy Supported by Gut Eating. Curr. Biol..

[18] Golubev A., Hanson A.D., Gladyshev V.N. (2018). A Tale of Two Concepts: Harmonizing the Free Radical and Antagonistic Pleiotropy Theories of Aging. Antioxid. Redox Signal..

[19] ATSDR (2012). Toxicological Profile for Manganese.

[20] Bouchard M.F., Sauve S., Barbeau B., Legrand M., Brodeur M.E., Bouffard T., Limoges E., Bellinger D.C., Mergler D. (2011). Intellectual impairment in school-age children exposed to manganese from drinking water. Environ. Health Perspect..

[21] Bowler R.M., Mergler D., Sassine M.P., Larribe F., Hudnell K. (1999). Neuropsychiatric effects of manganese on mood. Neurotoxicology.

[22] Claus Henn B., Ettinger A.S., Schwartz J., Tellez-Rojo M.M., Lamadrid-Figueroa H., Hernandez-Avila M., Schnaas L., Amarasiriwardena C., Bellinger D.C., Hu H. (2010). Early postnatal blood manganese levels and children’s neurodevelopment. Epidemiology.

[23] Kessissoglou D.P. (1995). Manganese-Proteins and -Enzymes and Relevant Trinuclear Synthetic Complexes. Bioinorganic Chemistry: An Inorganic Perspective of Life.

[24] Smith M.R., Fernandes J., Go Y.M., Jones D.P. (2017). Redox dynamics of manganese as a mitochondrial life-death switch. Biochem. Biophys. Res. Commun..

[25] Zhang S., Fu J., Zhou Z. (2004). In vitro effect of manganese chloride exposure on reactive oxygen species generation and respiratory chain complexes activities of mitochondria isolated from rat brain. Toxicol. In Vitro.

[26] Martinez-Finley E.J., Gavin C.E., Aschner M., Gunter T.E. (2013). Manganese neurotoxicity and the role of reactive oxygen species. Free Radic Biol. Med..

[27] Fernandes J., Hao L., Bijli K.M., Chandler J.D., Orr M., Hu X., Jones D.P., Go Y.M. (2017). From the Cover: Manganese Stimulates Mitochondrial H_2_O_2_ Production in SH-SY5Y Human Neuroblastoma Cells over Physiologic as well as Toxicologic Range. Toxicol. Sci..

[28] Aschner M., Aschner J.L. (1990). Manganese transport across the blood-brain barrier: Relationship to iron homeostasis. Brain Res. Bull..

[29] Gavin C.E., Gunter K.K., Gunter T.E. (1999). Manganese and calcium transport in mitochondria: Implications for manganese toxicity. Neurotoxicology.

[30] Pinsino A., Roccheri M.C., Costa C., Matranga V. (2011). Manganese interferes with calcium, perturbs ERK signaling, and produces embryos with no skeleton. Toxicol. Sci..

[31] Gavin C.E., Gunter K.K., Gunter T.E. (1992). Mn^2+^ sequestration by mitochondria and inhibition of oxidative phosphorylation. Toxicol. Appl. Pharmacol..

[32] Galvani P., Fumagalli P., Santagostino A. (1995). Vulnerability of mitochondrial complex I in PC12 cells exposed to manganese. Eur. J. Pharmacol..

[33] Fernandes J., Chandler J.D., Lili L.N., Uppal K., Hu X., Hao L., Go Y.M., Jones D.P. (2019). Transcriptome Analysis Reveals Distinct Responses to Physiologic versus Toxic Manganese Exposure in Human Neuroblastoma Cells. Front. Genet..

[34] Fernandes J., Chandler J.D., Liu K.H., Uppal K., Hao L., Hu X., Go Y.M., Jones D.P. (2019). Metabolomic Responses to Manganese Dose in SH-SY5Y Human Neuroblastoma Cells. Toxicol. Sci..

[35] Uppal K., Ma C., Go Y.M., Jones D.P., Wren J. (2018). xMWAS: A data-driven integration and differential network analysis tool. Bioinformatics.

[36] Yu T., Park Y., Johnson J.M., Jones D.P. (2009). apLCMS—adaptive processing of high-resolution LC/MS data. Bioinformatics.

[37] Uppal K., Soltow Q.A., Strobel F.H., Pittard W.S., Gernert K.M., Yu T., Jones D.P. (2013). xMSanalyzer: Automated pipeline for improved feature detection and downstream analysis of large-scale, non-targeted metabolomics data. BMC Bioinform..

[38] Love M.I., Huber W., Anders S. (2014). Moderated estimation of fold change and dispersion for RNA-seq data with DESeq2. Genome Biol..

[39] Blondel V.D., Guillaume J.L., Lambiotte R., Lefebvre E. (2008). Fast unfolding of communities in large networks. J. Stat. Mech. Theory Exp..

[40] Li S., Park Y., Duraisingham S., Strobel F.H., Khan N., Soltow Q.A., Jones D.P., Pulendran B. (2013). Predicting network activity from high throughput metabolomics. PLoS Comput. Biol..

[41] Dennis G., Sherman B.T., Hosack D.A., Yang J., Gao W., Lane H.C., Lempicki R.A. (2003). DAVID: Database for Annotation, Visualization, and Integrated Discovery. Genome Biol..

[42] Uppal K., Walker D.I., Jones D.P. (2017). xMSannotator: An R Package for Network-Based Annotation of High-Resolution Metabolomics Data. Anal. Chem..

[43] Schymanski E.L., Jeon J., Gulde R., Fenner K., Ruff M., Singer H.P., Hollender J. (2014). Identifying small molecules via high resolution mass spectrometry: Communicating confidence. Environ. Sci. Technol..

[44] Kanehisa M., Furumichi M., Tanabe M., Sato Y., Morishima K. (2017). KEGG: New perspectives on genomes, pathways, diseases and drugs. Nucleic Acids Res..

[45] Wishart D.S., Jewison T., Guo A.C., Wilson M., Knox C., Liu Y., Djoumbou Y., Mandal R., Aziat F., Dong E. (2013). HMDB 3.0—The Human Metabolome Database in 2013. Nucleic Acids Res..

[46] Pedley A.M., Benkovic S.J. (2017). A New View into the Regulation of Purine Metabolism: The Purinosome. Trends Biochem. Sci..

[47] Saarimaki-Vire J., Alitalo A., Partanen J. (2011). Analysis of Cdh22 expression and function in the developing mouse brain. Dev. Dyn..

[48] Harris L., Genovesi L.A., Gronostajski R.M., Wainwright B.J., Piper M. (2015). Nuclear factor one transcription factors: Divergent functions in developmental versus adult stem cell populations. Dev. Dyn..

[49] Bai M., Agnantis N.J., Skyrlas A., Tsanou E., Kamina S., Galani V., Kanavaros P. (2003). Increased expression of the bcl6 and CD10 proteins is associated with increased apoptosis and proliferation in diffuse large B-cell lymphomas. Mod. Pathol..

[50] Anneren C., Lindholm C.K., Kriz V., Welsh M. (2003). The FRK/RAK-SHB signaling cascade: A versatile signal-transduction pathway that regulates cell survival, differentiation and proliferation. Curr. Mol. Med..

[51] Thomas S.M., Brugge J.S. (1997). Cellular functions regulated by Src family kinases. Annu. Rev. Cell Dev. Biol..

[52] Imamura I., Watanabe T., Hase Y., Sakamoto Y., Fukuda Y., Yamamoto H., Tsuruhara T., Wada H. (1984). Histamine metabolism in patients with histidinemia: Determination of urinary levels of histamine, N tau-methylhistamine, imidazole acetic acid, and its conjugate(s). J. Biochem..

[53] Shi D., Caldovic L., Tuchman M. (2018). Sources and Fates of Carbamyl Phosphate: A Labile Energy-Rich Molecule with Multiple Facets. Biology.

[54] Maubach G., Schmadicke A.C., Naumann M. (2017). NEMO Links Nuclear Factor-kappaB to Human Diseases. Trends Mol. Med..

[55] Liu T., Zhang L., Joo D., Sun S.C. (2017). NF-kappaB signaling in inflammation. Signal Transduct. Target. Ther..

[56] Chong Z.Z., Shang Y.C., Hou J., Maiese K. (2010). Wnt1 neuroprotection translates into improved neurological function during oxidant stress and cerebral ischemia through AKT1 and mitochondrial apoptotic pathways. Oxidative Med. Cell. Longev..

[57] Williams M.E., Marubio L.M., Deal C.R., Hans M., Brust P.F., Philipson L.H., Miller R.J., Johnson E.C., Harpold M.M., Ellis S.B. (1994). Structure and functional characterization of neuronal alpha 1E calcium channel subtypes. J. Biol. Chem..

[58] Nyholt D.R., LaForge K.S., Kallela M., Alakurtti K., Anttila V., Farkkila M., Hamalainen E., Kaprio J., Kaunisto M.A., Heath A.C. (2008). A high-density association screen of 155 ion transport genes for involvement with common migraine. Hum. Mol. Genet..

[59] Ambrosini A., D’Onofrio M., Buzzi M.G., Arisi I., Grieco G.S., Pierelli F., Santorelli F.M., Schoenen J. (2017). Possible Involvement of the CACNA1E Gene in Migraine: A Search for Single Nucleotide Polymorphism in Different Clinical Phenotypes. Headache.

[60] Pascual J.M., Carceller F., Roda J.M., Cerdan S. (1998). Glutamate, glutamine, and GABA as substrates for the neuronal and glial compartments after focal cerebral ischemia in rats. Stroke.

[61] Kamal M.A., Mushtaq G., Greig N.H. (2015). Current Update on Synopsis of miRNA Dysregulation in Neurological Disorders. CNS Neurol. Disord. Drug Targets.

[62] Li L., Zhuang Y., Zhao X., Li X. (2018). Long Non-coding RNA in Neuronal Development and Neurological Disorders. Front. Genet..

[63] Stern M. (2017). Evidence that a mitochondrial death spiral underlies antagonistic pleiotropy. Aging Cell.

[64] Mauck K.E., Chesnais Q., Shapiro L.R. (2018). Evolutionary Determinants of Host and Vector Manipulation by Plant Viruses. Adv. Virus Res..

[65] Dai D.F., Chiao Y.A., Martin G.M., Marcinek D.J., Basisty N., Quarles E.K., Rabinovitch P.S. (2017). Mitochondrial-Targeted Catalase: Extended Longevity and the Roles in Various Disease Models. Prog. Mol. Biol. Transl..

[66] Plachez C., Lindwall C., Sunn N., Piper M., Moldrich R.X., Campbell C.E., Osinski J.M., Gronostajski R.M., Richards L.J. (2008). Nuclear factor I gene expression in the developing forebrain. J. Comp. Neurol..

[67] Pandya J.D., Nukala V.N., Sullivan P.G. (2013). Concentration dependent effect of calcium on brain mitochondrial bioenergetics and oxidative stress parameters. Front. Neuroenergetics.

[68] Debray F.G., Stumpfig C., Vanlander A.V., Dideberg V., Josse C., Caberg J.H., Boemer F., Bours V., Stevens R., Seneca S. (2015). Mutation of the iron-sulfur cluster assembly gene IBA57 causes fatal infantile leukodystrophy. J. Inherit. Metab. Dis..

[69] Chugunova A., Loseva E., Mazin P., Mitina A., Navalayeu T., Bilan D., Vishnyakova P., Marey M., Golovina A., Serebryakova M. (2019). LINC00116 codes for a mitochondrial peptide linking respiration and lipid metabolism. Proc. Natl. Acad. Sci. USA.

[70] Strittmatter P., Spatz L., Corcoran D., Rogers M.J., Setlow B., Redline R. (1974). Purification and properties of rat liver microsomal stearyl coenzyme A desaturase. Proc. Natl. Acad. Sci. USA.

[71] Wilkins H.M., Swerdlow R.H. (2016). Relationships Between Mitochondria and Neuroinflammation: Implications for Alzheimer’s Disease. Curr. Top. Med. Chem..

[72] Verri M., Pastoris O., Dossena M., Aquilani R., Guerriero F., Cuzzoni G., Venturini L., Ricevuti G., Bongiorno A.I. (2012). Mitochondrial alterations, oxidative stress and neuroinflammation in Alzheimer’s disease. Int. J. Immunopathol. Pharmacol..

[73] Peruzzotti-Jametti L., Pluchino S. (2018). Targeting Mitochondrial Metabolism in Neuroinflammation: Towards a Therapy for Progressive Multiple Sclerosis. Trends Mol. Med..

[74] Bavner A., Shafaati M., Hansson M., Olin M., Shpitzen S., Meiner V., Leitersdorf E., Bjorkhem I. (2010). On the mechanism of accumulation of cholestanol in the brain of mice with a disruption of sterol 27-hydroxylase. J. Lipid Res..

[75] Nie S., Chen G., Cao X., Zhang Y. (2014). Cerebrotendinous xanthomatosis: A comprehensive review of pathogenesis, clinical manifestations, diagnosis, and management. Orphanet. J. Rare Dis..

[76] Repa J.J., Lund E.G., Horton J.D., Leitersdorf E., Russell D.W., Dietschy J.M., Turley S.D. (2000). Disruption of the sterol 27-hydroxylase gene in mice results in hepatomegaly and hypertriglyceridemia. Reversal by cholic acid feeding. J. Biol. Chem..

[77] Balcerczyk A., Bartosz G. (2003). Thiols are main determinants of total antioxidant capacity of cellular homogenates. Free Radic Res..

[78] Go Y.M., Orr M., Jones D.P. (2013). Actin cytoskeleton redox proteome oxidation by cadmium. Am. J. Physiol. Lung Cell. Mol. Physiol..

[79] Rosslenbroich V., Dai L., Baader S.L., Noegel A.A., Gieselmann V., Kappler J. (2005). Collapsin response mediator protein-4 regulates F-actin bundling. Exp. Cell Res..

[80] Shapovalova Z., Tabunshchyk K., Greer P.A. (2007). The Fer tyrosine kinase regulates an axon retraction response to Semaphorin 3A in dorsal root ganglion neurons. BMC Dev. Biol..

[81] Larti F., Kahrizi K., Musante L., Hu H., Papari E., Fattahi Z., Bazazzadegan N., Liu Z., Banan M., Garshasbi M. (2015). A defect in the CLIP1 gene (CLIP-170) can cause autosomal recessive intellectual disability. Eur. J. Hum. Genet..

[82] Sies H., Jones D.P. (2020). Reactive oxygen species (ROS) as pleiotropic physiological signalling agents. Nat. Rev. Mol. Cell Biol..

[83] Lambeth J.D. (2007). Nox enzymes, ROS, and chronic disease: An example of antagonistic pleiotropy. Free Radic Biol. Med..

[84] Neubauer J.A., Sunderram J. (2004). Oxygen-sensing neurons in the central nervous system. J. Appl. Physiol..

[85] Uppal K., Walker D.I., Liu K., Li S., Go Y.M., Jones D.P. (2016). Computational Metabolomics: A Framework for the Million Metabolome. Chem. Res. Toxicol..

[86] Hu X., Go Y.M., Jones D.P. (2020). Omics Integration for Mitochondria Systems Biology. Antioxid. Redox Signal..

[87] Serhan C.N., Levy B.D. (2018). Resolvins in inflammation: Emergence of the pro-resolving superfamily of mediators. J. Clin. Investig..

