# Supplementary material for: Antagonistic Interactions in Mitochondria ROS Signaling Responses to Manganese
Source: Antioxidants (Basel). 2023 Mar 25;12(4):804. doi: 10.3390/antiox12040804 (PMC10134992; doi:10.3390/antiox12040804)
Supplement: Supplementary file 1 [file antioxidants-12-00804-s001.zip › Figure S1.pptx]

## Slide 1
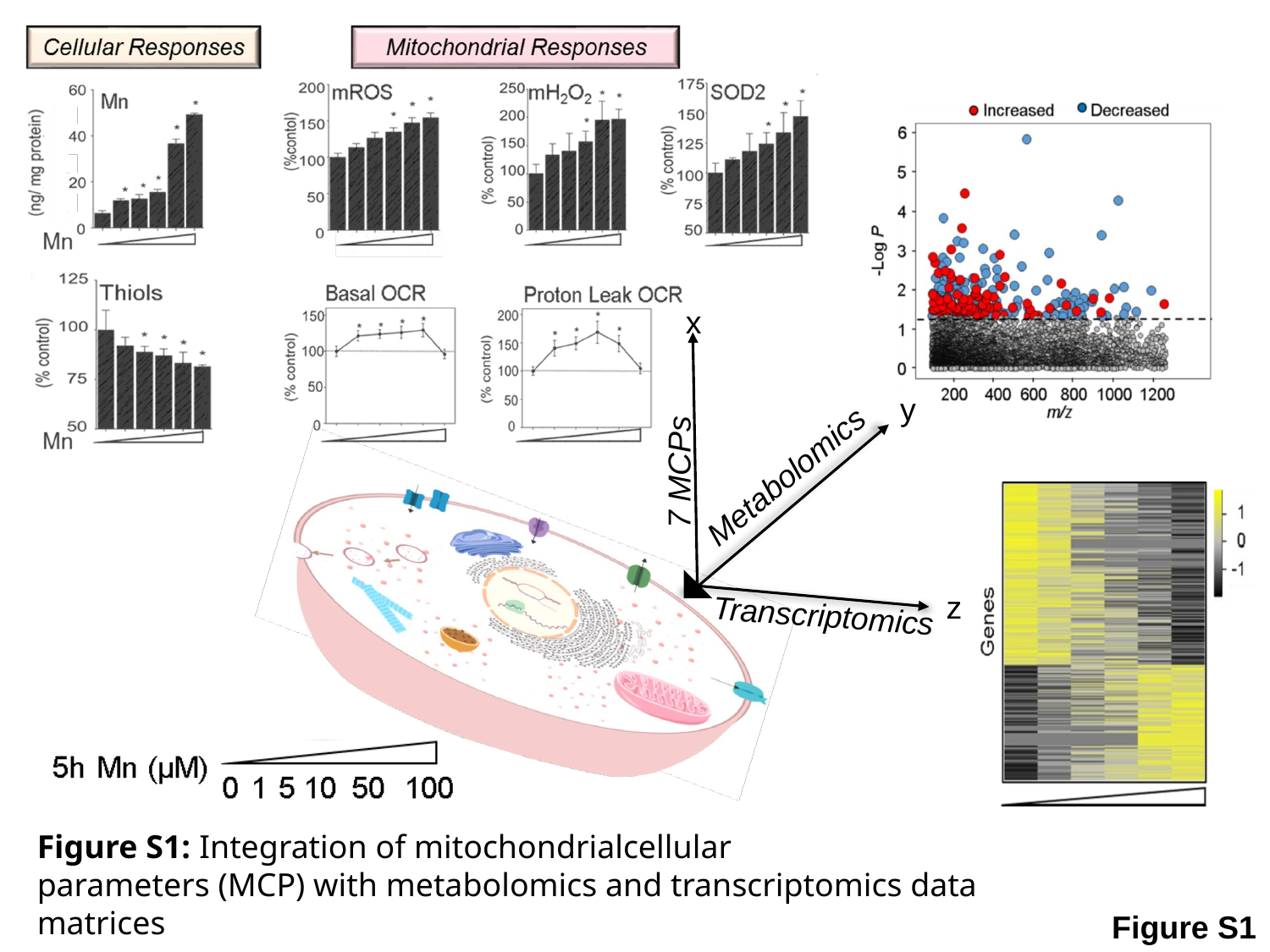

x
y
Metabolomics
 7 MCPs
z
Transcriptomics
Figure S1: Integration of mitochondrialcellular
parameters (MCP) with metabolomics and transcriptomics data matrices
Figure S1
