# Supplementary material for: Antagonistic Interactions in Mitochondria ROS Signaling Responses to Manganese
Source: Antioxidants (Basel). 2023 Mar 25;12(4):804. doi: 10.3390/antiox12040804 (PMC10134992; doi:10.3390/antiox12040804)
Supplement: Supplementary file 1 [file antioxidants-12-00804-s001.zip › Figure S2.pptx]

## Slide 1
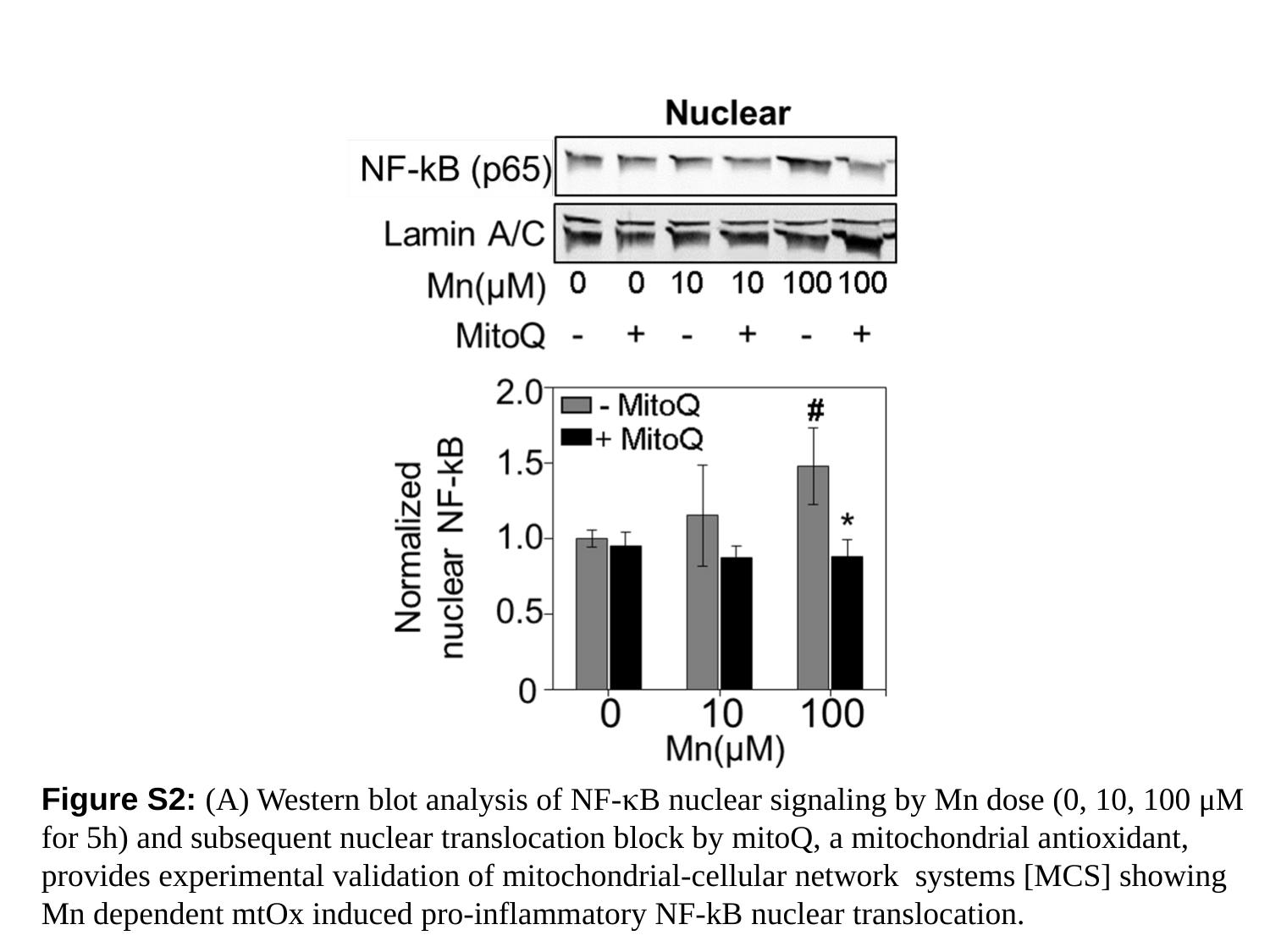

Figure S2: (A) Western blot analysis of NF-κB nuclear signaling by Mn dose (0, 10, 100 μM for 5h) and subsequent nuclear translocation block by mitoQ, a mitochondrial antioxidant, provides experimental validation of mitochondrial-cellular network systems [MCS] showing Mn dependent mtOx induced pro-inflammatory NF-kB nuclear translocation.
