# Supplementary material for: Antagonistic Interactions in Mitochondria ROS Signaling Responses to Manganese
Source: Antioxidants (Basel). 2023 Mar 25;12(4):804. doi: 10.3390/antiox12040804 (PMC10134992; doi:10.3390/antiox12040804)
Supplement: Supplementary file 1 [file antioxidants-12-00804-s001.zip › Figure S3.pptx]

## Slide 1
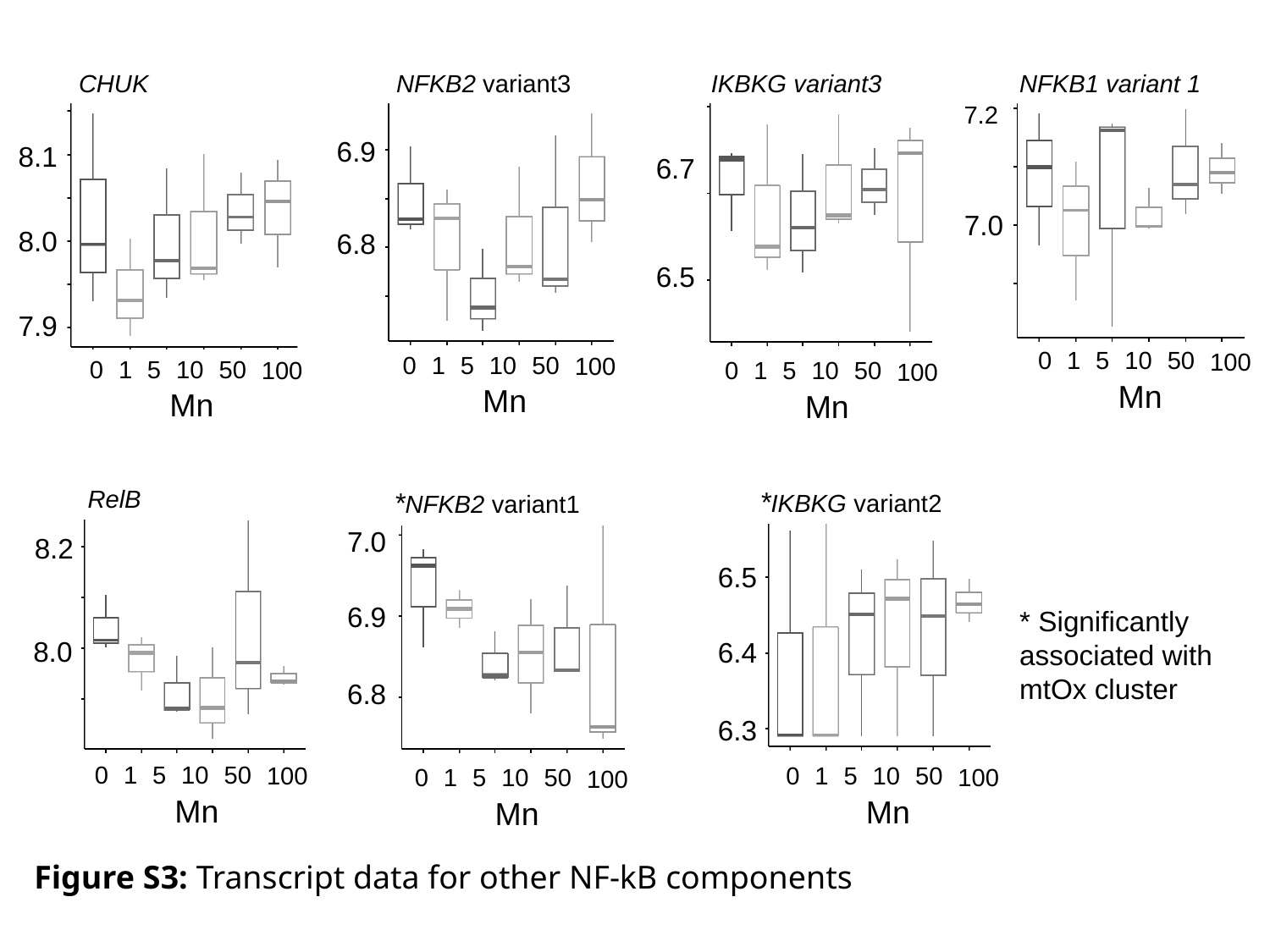

CHUK
NFKB2 variant3
IKBKG variant3
NFKB1 variant 1
7.2
6.9
8.1
6.7
7.0
8.0
6.8
6.5
7.9
0
1
5
10
50
100
0
1
5
10
50
100
0
1
5
10
50
0
1
5
10
50
100
100
Mn
Mn
Mn
Mn
*IKBKG variant2
RelB
*NFKB2 variant1
7.0
8.2
6.5
6.9
* Significantly associated with mtOx cluster
8.0
6.4
6.8
6.3
0
1
5
10
50
0
1
5
10
50
100
0
1
5
10
50
100
100
Mn
Mn
Mn
Figure S3: Transcript data for other NF-kB components
