# Supplementary material for: Antagonistic Interactions in Mitochondria ROS Signaling Responses to Manganese
Source: Antioxidants (Basel). 2023 Mar 25;12(4):804. doi: 10.3390/antiox12040804 (PMC10134992; doi:10.3390/antiox12040804)
Supplement: Supplementary file 1 [file antioxidants-12-00804-s001.zip › Text S1.pdf]

**Text S1: ABBREVIATIONS**

Mn, manganese

SOD2, mitochondrial superoxide dismutase

mt$H_2O_2$, mitochondrial hydrogen peroxide

mtROS, mitochondrial reactive oxygen species

bOCR, basal oxygen consumption rate

pOCR, proton leak dependent oxygen consumption rate

mtOCR, mitochondrial oxygen consumption rate (bOCR and pOCR combined)

SH-SY5Y, human neuroblastoma cell line

xMWAS, a software for data integration, network visualization, clustering, and differential network analysis

xMSannotator, R Package for Network-Based Annotation of High-Resolution Metabolomics Data

FYN, FYN proto-oncogene, Src family tyrosine kinase

FRK, fyn-related Src family tyrosine kinase

CHD22, cadherin 22, type 2

PAICS, phosphoribosylaminoimidazole carboxylase and succinocarboxamide synthetase

NFIB, nuclear factor I/B

BCL6, B-cell CLL/lymphoma 6

WNT1, wingless-type MMTV integration site family

IKBKG, inhibitor of kappa light polypeptide gene enhancer in B-cells, kinase gamma

MAPK8, mitogen-activated protein kinase 8

HNMT, histamine N-methyltransferase

LIPG, lipase, endothelial

CPT1A, carnitine palmitoyltransferase 1A

DPYSL3, dihydropyrimidinase-like 3

FER, fer (fps/fes related) tyrosine kinase

GPX8, glutathione peroxidase 8

CLRN3, clarin 3

UBB, ubiquitin B

UPP1, uridine phosphorylase 1

CACNA1E, calcium channel, voltage-dependent, R type, alpha 1E subunit

KCNC4, potassium channel, voltage gated Shaw related subfamily C, member 4

IBA57, iron-sulfur cluster assembly homolog

SIRT6, sirtuin 6

CYB5R3, cytochrome b5 reductase 3
